# Differences in Sleep Spindle Characteristics Between Children With Narcolepsy and Those With Other Causes of Subjective Sleepiness

**DOI:** 10.1111/jsr.70415

**Published:** 2026-07-28

**Authors:** Marisha Shetty, Nushrika Kadam Kishore, Georgina Plunkett, Lauren C. Nisbet, Margot J. Davey, Gillian M. Nixon, Lisa M. Walter, Rosemary S. C. Horne

**Affiliations:** ^1^ Department of Paediatrics Monash University Melbourne Australia; ^2^ Melbourne Children's Sleep Centre, Monash Children's Hospital Melbourne Australia

**Keywords:** excessive daytime sleepiness, hypersomnolence, paediatric, sleep

## Abstract

Central disorders of hypersomnolence are characterised by excessive daytime sleepiness. There has been growing interest in analysing sleep micro‐architecture, to further understand how sleep is affected by these conditions. We investigated if sleep spindle activity differed between children with narcolepsy, idiopathic hypersomnolence (IH) and subjectively sleepy children with a non‐diagnostic multiple sleep latency test, and individually age‐ and sex‐matched controls. Twenty eight children with narcolepsy, 11 with IH and 26 with subjective sleepiness (aged 6–19 years) were included. Spindle activity was characterised as spindle density (number of spindles/min) and intensity (density × average duration) of central (C4) and frontal (F4) EEG electrodes for Slow and Fast spindles and combined (Slow&Fast) across the sleep period and also when separated into NREM sleep periods (NREMP) across the night. C4 Slow and Fast and F4 Slow spindle duration was longer in children with narcolepsy compared to their controls (*p* < 0.05 for all comparisons) and to those with subjective sleepiness (*p* < 0.05). In the children with narcolepsy and subjective sleepiness C4 spindle intensity was greater (*p* < 0.05 for both groups) compared to controls. C4 spindle density and intensity were lower at the beginning compared to the end of the night in the children with narcolepsy and subjective sleepiness (*p* < 0.05 for both groups). There were no relationships between spindle activity and parental reports of daytime sleepiness. Spindle characteristics differed between children with narcolepsy compared to controls and to children with undiagnosed subjective sleepiness. Our findings may aid in distinguishing between groups of children referred for assessment of excessive daytime sleepiness.

## 1 Introduction

Central disorders of hypersomnolence are characterised by excessive daytime sleepiness (EDS) that is not explained by poor sleep schedules, insufficient sleep or underlying medical conditions. Included in this category of sleep disorders are narcolepsy and idiopathic hypersomnia (IH). There has been growing interest in analysing sleep micro‐architecture, to further understand how sleep is affected by these conditions (Weiner and Dang‐Vu 2016). Previously, using spectral analysis of the electro‐encephalogram (EEG) during an overnight polysomnographic (PSG) study we identified that relative Theta power was higher in the narcolepsy compared with the subjectively sleepy group during wake (*p* < 0.001), N2 (*p* < 0.01), N3 (*p* < 0.05) and total sleep (*p* < 0.01) and relative Delta power during total sleep was lower in both the narcolepsy (*p* < 0.05) and IH (*p* < 0.05) groups compared to others with subjective sleepiness who had a non‐diagnostic multiple sleep latency test (MSLT). We suggested that these sleep micro‐architecture features may provide insights into the pathophysiology of these conditions (Plunkett et al. 2024). There has been growing interest in examining specific features of the EEG, such as sleep spindles, which play a primary role in protecting the sleeping brain from external sensory stimuli and can serve as markers of sleep integrity (Dang‐Vu et al. 2010; Dang‐Vu et al. 2015; Saletin et al. 2013). The finding that increased spindles may enhance sleep stability and therefore protect sleep from disruption is especially important in the context of narcolepsy as patients characteristically have disrupted nocturnal sleep (Lee et al. 2024; Maski et al. 2021).

Sleep spindles are a hallmark waveform of N2 sleep and represent an oscillating electrical potential in the brain. Spindles are generated in the thalamus and synchronised in the cortex and therefore reflect thalamocortical activity (Steriade 1999). They have a characteristic frequency in the sigma range of 11–16 Hz and last from 0.5 to 3 s in duration. On the EEG, spindles are visually identified as sinusoidal waves that have a ‘crescendo‐decrescendo’ pattern (De Gennaro and Ferrara 2003). Sleep spindles can be divided into two distinct types based on their frequency: slow spindles (< 12 Hz) that occur maximally over frontal regions and fast spindles (≥ 12 Hz) which dominate in central and parietal regions (Mölle et al. 2011). In addition, frontal and centroparietal spindles exhibit different maturational patterns, with the frequency of centroparietal spindles being relatively unchanged with age over childhood and adolescence, while frontal spindles become more prominent with age, with an increase in frequency around puberty (Ricci et al. 2021), coinciding with the peak age of onset of hypersomnolence disorders (Bruni 2023). As it has been suggested that slow and fast spindles serve distinct functional roles and may be differentially affected in neuropsychiatric disorders, it is important that these spindle types are separated (Cox et al. 2017).

Previous studies examining sleep spindles in patients with narcolepsy and IH have studied small populations that mainly included adults, using differing methods of analysis (Bove et al. 1994; Christensen et al. 2017; DelRosso et al. 2014). Bove et al. identified spindles manually while Christensen et al. and DelRosso et al. used different automated methods of identification (Bove et al. 1994; Christensen et al. 2017; DelRosso et al. 2014). These studies have reported differing findings, with one study reporting higher spindle densities in patients with narcolepsy and IH compared to controls (Bove et al. 1994), while another found no difference between groups (Christensen et al. 2017). Two studies have reported that patients with IH had higher spindle densities than those with narcolepsy (Bove et al. 1994; DelRosso et al. 2014). Studies have also reported differences in spindle density across the night; however, the methods of analysis have differed between the studies. Bove et al. reported people with narcolepsy had the highest spindle density at the beginning of the night while those with IH had the highest spindle density towards the end of the night (Bove et al. 1994). In contrast, DelRosso et al. reported higher spindle densities at the end of the night in both narcolepsy and IH patients (DelRosso et al. 2014). The most recent study compared automatically detected sleep spindle characteristics between children and adolescents (8–21 years old) with narcolepsy Type 1 (NT1) and healthy controls (Maski et al. 2025). They found that sleep spindle characteristics were similar between groups, with the exception that the patients with NT1 had shorter sleep spindles compared to the healthy controls.

The inconsistencies in the findings of previous studies examining spindle characteristics in subjects with narcolepsy and IH may be due to these studies combining both adults and children. In addition, previous studies have not separated spindles by their frequency or location, which is important given that these characteristics are likely to be affected differently by different sleep disorders (Weiner and Dang‐Vu 2016). Therefore, this study aimed to compare sleep spindle characteristics (duration, density and intensity), frequency (Fast and Slow) and location (central and frontal) between children and adolescents with MSLT‐confirmed narcolepsy and IH, those with an abnormal but non‐diagnostic MSLT (termed subjective sleepiness) and matched controls during total NREM sleep and in individual NREM periods across the night. We further aimed to relate spindle density and intensity with measures of daytime sleepiness assessed by questionnaire. We hypothesised that children with narcolepsy and IH would exhibit increased spindle duration, density and intensity compared to those with subjective sleepiness and their matched controls and that spindle density and intensity would be more marked at the end of the night. Further, we hypothesised that spindle activity would be positively correlated with reported daytime sleepiness.

## 2 Methods

Ethical approval for this study was provided from the Monash Health Human Research Ethics Committee (04161C). Written informed consent was obtained from parents to use their child's data for research. All children having an overnight polysomnographic (PSG) study followed by a multiple sleep latency test (MSLT) for investigation of EDS between May 2010 and December 2023 were eligible. Children were excluded from analysis if they had an obstructive apnoea‐hypopnoea index (OAHI) > 5 events/h, or were using any medications with the potential to affect REM sleep on the day of the MSLT (e.g., were unable to cease selective serotonin reuptake inhibitors for at least 2 weeks before the study).

Published diagnostic criteria were used to define narcolepsy and idiopathic hypersomnia (American Academy of Sleep Medicine 2014; Krahn et al. 2021; Littner et al. 2005). Children with narcolepsy had a mean sleep latency ≤ 8 min (an average of the time taken to fall asleep over 4 or 5 naps on their MSLT) AND ≥ 2 sleep onset REM periods (SOREMPs) within the first 15 min of falling asleep (on MSLT or overnight PSG). Since 2014 this can include the presence of a nocturnal SOREM (a REM period within the first 15 min of initial sleep onset on the overnight PSG) (American Academy of Sleep Medicine 2014). As we did not have cerebrospinal fluid measurements of hypocretin‐1 levels or a reliable history of cataplexy to distinguish between children with NT1 and NT2, our narcolepsy group was likely a combination of the two. Children with IH had a mean sleep latency ≤ 8 min AND < 2 SOREMPs. A third group of children who did not meet the MSLT criteria for either narcolepsy or IH was also included: the subjectively sleepy group. This group had a comparable Epworth Sleepiness Scale Child and Adolescent questionnaire (ESS‐CHAD) score to the primary disorders of hypersomnolence groups, but did not meet the criteria for a positive MSLT based on current standard criteria. Some of these children may have an evolving primary disorder of hypersomnolence (Nisbet et al. 2024).

Control children who had been referred for PSG for reasons other than assessment of EDS, primarily for the assessment of sleep disordered breathing, who underwent PSG without MSLT within the same timeframe were identified from the Melbourne Children's Sleep Centre research database and individually matched to each child with EDS, with the selected control being the child closest in age and OAHI and, where possible, of the same sex.

Results of differences in autonomic cardiovascular control between these groups of children (Francis et al. 2025), spectral analysis of the EEG (Plunkett et al. 2024) and sleep state transitions (Lee et al. 2024) have previously been published.

Prior to the PSG study, height and weight were measured and body mass index (BMI) z‐score was calculated. Obesity was defined as ≥ 95th percentile (BMI z scores ≥ 1.65) and overweight as ≥ 85th percentile (BMI z‐scores ≥ 1.04). Parents completed the Epworth Sleepiness Scale for Children and Adolescents (ESS‐CHAD) (Janssen et al. 2017).

All PSG studies were conducted by trained paediatric sleep scientists in accordance with the guidelines set out by the American Academy of Sleep Medicine (AASM) for paediatric sleep studies, current at the time the study (Berry et al. 2020; Berry et al. 2012; Iber et al. 2007). Electrophysiological signals were recorded using a commercially available PSG system (E‐Series or Grael, Compumedics, Melbourne, Australia) with signals recorded at a sampling rate of 512 Hz. The montage included EEG (Cz, F3‐M2, F4‐M1, C3‐M2, C4‐M1, O1‐M2, O2‐M1), right and left electrooculogram (EOG), submental electromyogram (EMG), left and right anterior tibialis muscle EMG and electrocardiogram (ECG). Respiratory characteristics were captured using abdominal and thoracic respiratory plethysmography (Pro‐Tech zRIP Effort Sensor, Pro‐Tech Services Inc., Mukilteo, WA), oronasal thermistor, nasal pressure and transcutaneous carbon dioxide (TcCO_2_), (TCM4/40, Radiometer, Denmark, Copenhagen or Sentec, Therwil, Switzerland). Peripheral oxygen saturation (SpO_2_) was measured using Bitmos GmbH (Bitmos, Dusseldorf, Germany), which uses Masimo signal extraction technology for signal processing or Masimo Radical 7 (Masimo, Irving, CA), with both devices set to a 2‐s averaging time.

### 2.1 Sleep and Respiratory Analysis

All PSG studies were scored manually in 30 s epochs for sleep stages (N1, N2, N3 and REM) and arousals, and in 2 min epochs for respiratory events > 2 breaths in duration, by trained paediatric sleep scientists using Compumedics ProFusion software according to AASM paediatric guidelines at the time of the study (Berry et al. 2020; Berry et al. 2012; Iber et al. 2007). An obstructive apnoea was defined as the cessation of airflow in association with ongoing respiratory effort; an obstructive hypopnoea was defined as ≥ 30% decrease in nasal pressure signal amplitude, associated with increased work of breathing and an arousal or ≥ 3% decrease in oxygen saturation; a central apnoea was defined as cessation of airflow without inspiratory effort lasting either ≥ 20 s or at least the duration of two breaths and associated with an arousal or ≥ 3% oxygen desaturation; and a central hypopnoea as ≥ 30% decrease in nasal pressure signal amplitude with reduced inspiratory effort throughout the entire duration of the event. A mixed apnoea was defined if an event was associated with absent respiratory effort during one portion of the event and the presence of obstructed inspiratory efforts in another portion, regardless of which portion came first (Berry et al. 2012). The OAHI, defined as the total number of obstructive apnoeas, mixed apnoeas and obstructive hypopnoeas per hour of total sleep time (TST) was used to define SDB severity. Other respiratory parameters included the respiratory disturbance index (RDI), defined as the total number of respiratory events including obstructive and central apnoeas, mixed apnoeas, obstructive and central hypopnoeas; the REM RDI; the arousal index (ArI), defined as the number of cortical EEG arousals per hour of TST; the central apnoea hypopnoea index (CAHI), defined as the number of central apnoeas and hypopnoeas per hour of TST; and the. SpO_2_ nadir, defined as the lowest oxygen saturation associated with a respiratory event.

### 2.2 Sleep Macro‐Architecture Analysis

For studies recorded prior to 2012, NREM 3 and NREM 4 sleep were combined into N3. Standard measures of sleep quality were calculated for each participant and included the following parameters. The duration of each sleep stage (N1, N2, N3, REM) was expressed as a % of TST. Wake after sleep onset (WASO) was calculated as the percentage of time awake during the sleep period time (SPT), defined as the amount of time in minutes from sleep onset until lights on at the end of the study, including all periods of wake in between. TST was defined as SPT excluding all periods of wake. Time in bed (TIB) was defined as the time between lights off and lights on. Sleep latency was defined as the period from lights off to the first three consecutive epochs of N1 sleep or an epoch of any other stage. Sleep efficiency was defined as the ratio of TST to TIB.

### 2.3 Sleep Spindles Analysis

Sleep spindles were identified manually by 1 investigator (MS) using Compumedics Profusion 3.0 software (Melbourne, Australia) (Shetty et al. 2024). Spindles were identified from the C4‐M1 and F4‐M1 EEG channels during N2 and N3 sleep from raw EEG signals recorded using a band‐pass filter of 0.3–100 Hz and a sampling frequency of 512 Hz. Each 30 s epoch was studied with the EEG gain set to 25 μV/mm, the high pass band filtered at 0.3 Hz and low pass band filtered at 35 Hz (Shetty et al. 2023). Spindles were categorised according to criteria reported by Chatburn et al. where identified spindles were required to be relatively equal in amplitude and with waves of a consistent frequency and a narrow conical shape with a duration of > 0.5 s (Chatburn et al. 2013). Candidate spindles were not included if the amplitudes of the waves were not consistent and the waves were not conical, as if waves were too broad, the minimum of 5 peaks within 0.5 s would not be met and if too variable then this was most likely to be artefact. Waveforms that could otherwise be defined as a vertex or sawtooth wave or of highly variable amplitudes or frequencies were also excluded (Chatburn et al. 2013). Spindles were then categorised and labelled as either being F4 or C4 Slow (< 12 Hz) or F4 or C4 Fast (≥ 12 Hz) spindles, and F4 or C4 Slow and Fast spindles combined (Slow&Fast) to provide the total number of spindles and allow comparison with previous studies. Slow spindles consisted of 5–6 peaks while fast spindles had ≥ 7 peaks within 0.5 s. Broken spindles (spindles within 1 s of each other) were counted as one spindle (Chatburn et al. 2013).

Total spindle number was the total number of each category of spindle found in N2 and N3 sleep. The duration of every individual spindle was calculated by Profusion, and when exported, verified to have a duration > 0.5 s before calculating the average duration for each spindle category for each child. Spindle density was calculated as the number of spindles per minute of N2 or N3 sleep; spindle intensity was calculated as the product of spindle density and average spindle duration. The majority of spindles were identified in N2 sleep as expected, but as including spindles from N3 increased the number of children with spindles in the various categories, data are presented with spindles from N2 and N3 combined. Sleep spindle characteristics across the night were assessed in each NREM period (NREMP) as determined from sleep stage scored data. A NREM period was defined as the succession of N2 or N3 of > 15 min duration that was terminated by either one 30 s epoch of REM or a period of wakefulness of at least 5 min duration (Plunkett et al. 2024). Only the first four NREM periods across the night were analysed as the majority of children had at least four NREM periods.

### 2.4 Statistical Analysis

All statistical analyses were performed with Sigma Plot (Systat Software Inc. Version 14.5). Data were first tested for normality and equal variance. Demographic, sleep and respiratory characteristics were compared between groups with one‐way analysis of variance (ANOVA) with Bonferroni post hoc tests if normally distributed and a Kruskal Wallis one‐way ANOVA on ranks with Dunn's post hoc tests if not. Differences between spindle characteristics (duration, density and intensity) were not normally distributed and were compared between the clinical groups (narcolepsy, IH and subjectively sleepy) and their controls (total of six groups) with Kruskal Wallis one‐way ANOVA on ranks with Dunn's post hoc tests for each spindle type. Differences between F4 and C4 spindle indices were compared with Wilcoxon Signed Rank tests. Differences in F4 and C4 Slow and Fast spindles combined spindle characteristics between the clinical groups and their controls in each NREM period across the night were also compared with Kruskal Wallis one‐way ANOVA on ranks with Dunn's post hoc tests. Associations between spindle activity indices and ESS‐CHAD scores were tested using Spearman rank‐order correlations. Data are presented as median and interquartile range (IQR) and a *p* value of < 0.05 was taken as statistically significant.

## 3 Results

Eighty one children with EDS aged 6–19 years were eligible; seven children were excluded as they had an OAHI > 5 events/h and nine were excluded as they were taking medications which would affect REM sleep on the night of the study. Of the 65 children included, 28 children were diagnosed with narcolepsy, 11 with IH and 26 were in the subjectively sleepy group. The demographic details for the clinical children and their matched controls are presented in Table 1. There were no differences between the groups for age, BMI z‐score or % overweight or obese. Children with narcolepsy and IH had elevated scores on the ESS‐CHAD compared to their controls (*p* < 0.001 for both), but were not significantly different from the subjectively sleepy group.

**TABLE 1:** Demographic and questionnaire characteristics for the narcolepsy, idiopathic hypersomnia and subjectively sleepy children and their matched controls. Values are median and IQR.

	Narcolepsy	Narcolepsy controls	IH	IH controls	Subjectively sleepy	Subjectively sleepy controls
*N*	28	28	11	11	26	26
Sex	12F/16M	11F/17M	7F/4M	7F/4M	18F/8M	15F/11M
Age	14.4 (11.9–16.3)	13.9 (11.1–16.0)	16.1 (14.9–16.3)	15.4 (14.5–16.3)	15.6 (13.9–17.3)	15.8 (13.5–17.1)
BMI z‐score	0.9 (0.3–1.4)	0.3 (−0.7–0.6)	0.8 (0.2–0.9)	0.5 (0.0–0.8)	0.6 (0.2–1.5)	0.7 (0.0–1.4)
% Obese	14	7	0	0	18	18
% Overweight	25	14	9	18	18	7
ESS‐CHAD	15 (13–16)***	7 (5–10)	15 (14–17)^†††^	6 (3–9)	13 (11–14)	9 (5–11)

*** *p* < 0.001 narcolepsy versus narcolepsy controls.

^†††^ *p* < 0.001 idiopathic hypersomnia versus idiopathic hypersomnia controls.

### 3.1 Sleep Macro‐Architecture

Sleep and respiratory characteristics for the groups are presented in Table 2. TST was significantly longer in the narcolepsy, IH and subjectively sleepy groups compared to their controls. Sleep latency and REM latency were significantly shorter in the narcolepsy group compared to their control group. Sleep latency was also shorter in the narcolepsy group compared to the subjectively sleepy group. The children with narcolepsy spent less time in NREM sleep and more time in REM sleep compared to their controls. Arousal Index was higher in both the narcolepsy and IH groups compared to their respective controls (*p* < 0.05 for both).

**TABLE 2:** Sleep and respiratory characteristics on overnight polysomnography of the narcolepsy, idiopathic hypersomnia and subjectively sleepy children and their matched controls. Values are median and IQR.

	Narcolepsy	Narcolepsy controls	IH	IH controls	Subjectively sleepy	Subjectively sleepy controls
*N*	28	28	11	11	26	26
Total sleep time (min)	495 (463–521)***	399 (384–425)	475 (446–504)^††^	389 (344–401)	474 (446–509)^‡‡‡^	413 (354–437)
Sleep latency (min)	7 (2–10)***^##^	27 (7–38)	12 (8–33)	18 (13–33)	17 (12–30)	25 (14–39)
REM latency (min)	66 (1–91)**	136 (102–179)	96 (69–165)	139 (107–237)	112 (79–171)	168 (127–207)
Sleep efficiency (%)	88.6 (83.4–91.4)	84.3 (79.9–89.6)	83.3 (80.2–89.2)	83.6 (74.3–89.7)	85.5 (80.6–91.1)	84.1 (71.2–90.5)
WASO (%)	10.5 (6.8–15.5)	9.5 (5.0–15.0)	12.0 (5.5–13.5)	10.0 (9.0–20.0)	7.5 (4.0–14.0)	9.5 (4.3–21.6)
N1 %TST	6.8 (4.7–13.8)	6.9 (4.9–8.9)	6.0 (3.7–14.2)	6.9 (3.9–8.1)	5.0 (3.9–9.4)	6.9 (4.9–9.9)
N2 %TST	47.0 (37.3–52.2)	50.4 (48.0–55.6)	53.0 (45.5–58.0)	54.5 (49.4–61.0)	49.2 (44.8–54.8)	51.2 (47.2–56.4)
N3 %TST	21.1 (16.7–25.9)	23.7 (19.3–27.3)	18.0 (7.9–20.5)	19.0 (16.0–24.0)	20.8 (16.0–24.0)	22.5 (18.1–25.0)
NREM sleep %TST	74.2 (71.4–78.0)***	81.9 (76.1–85.2)	77.0 (75.6–82.8)	80.1 (76.6–85.9)	78.0 (76.3–79.5)	82.5 (76.9–86.2)
REM sleep %TST	25.4 (21.4–28.1)***	18.1 (14.8–22.5)	22.0 (16.7–24.4)	19.9 (13.7–23.5)	21.1 (20.5–23.0)	17.5 (12.5–22.9)
Duration NREMP1 (min)	82 (70–125)	100 (66–135)	78 (62–111)	75 (62–121)	81 (56–125)	128 (65–158)
Duration NREMP2 (min)	67 (51–71)	74 (61–85)	82 (63–84)	73 (45–90)	66 (57–83)	75 (41–89)
Duration NREMP3 (min)	66 (47–80)	62 (47–73)	63 (52–71)	69 (68–87)	67 (60–79)	64 (48–70)
Duration NREMP4 (min)	62 (44–75)	67 (57–71)	69 (64–80)	53 (48–72)	64 (51–73)	44 (35–65)
OAHI (events/h)	0.2 (0.0–0.7)	0.4 (0.0–0.8)	0.3 (0.0–0.8)	0.0 (0–0.2)	0.4 (0.0–1.5)	0.1 (0.0–1.1)
CAHI (events/h)	0.5 (0.2–0.8)	0.6 (0.2–1.5)	0.5 (0.1–0.9)	0.3 (0.1–1.1)	0.6 (0.2–1.0)	0.6 (0.1–1.1)
RDI (events/h)	2.3 (1.0–3.4)	1.6 (1.0–2.3)	1.6 (0.6–2.8)	0.5 (0.3–2.8)	2.4 (0.9–3.5)	1.7 (0.6–3.8)
REM RDI (events/h)	2.6 (1.4–5.2)	1.5 (0.5–2.5)	2.2 (1.3–3.2)	0.0 (0.0–0.6)	2.0 (0.6–3.9)	1.3 (0.0–2.5)
Arousal Index (events/h)	14.3 (12.0–17.2)*	11.0 (9.0–12.3)	17.0 (11.9–18.5)^†^	9.1 (6.9–12.0)	12.0 (9.6–17.1)	10.8 (8.9–13.1)
SpO_2_ Nadir (%)	94.0 (92.0–95.0)	93.0 (91.0–94.0)	95.0 (92.5–96.0)	94.0 (90.0–95.0)	93.0 (91.0–95.0)	93.0 (91.0–95.0)

*Note:* *p < 0.05; **p < 0.01; ***p < 0.001 Narcolepsy versus narcolepsy controls. †*p* < 0.05; ††*p* < 0.01 Idiopathic hypersomnia versus idiopathic hypersomnia controls. ‡‡‡ *p* < 0.001 subjectively sleepy versus subjectively sleep controls. ##*p* < 0.01 Narcolepsy versus subjectively sleepy.

### 3.2 Differences in Spindle Indices Between Children With Narcolepsy, IH and Subjective Sleepiness and Their Controls

Comparison of spindle duration is presented in Figure 1. Spindle duration was longer for F4 Slow (median 0.572 vs. 0.522, *p* = 0.007) and C4 Slow (median 0.562 vs. 0.524, *p* = 0.009) and F4 (median 0.570 vs. 0.522, *p* = 0.018) and C4 (median 0.575 vs. 0.526, 0.007) Slow&Fast spindles in the children with narcolepsy compared to the children with subjective sleepiness. In the children with narcolepsy, spindle duration was longer for both F4 (median 0.572 vs. 0.525, *p* = 0.046) and C4 (median 0.562 vs. 0.522, *p* = 0.009) Slow, C4 Fast (median 0.620 vs. 0.525, *p* = 0.007) and F4 (median 0.570 vs. 0.522, *p* = 0.041) and C4 (median 0.575 vs. 0.525, *p* = 0.015) Fast&Slow spindles compared to controls. There were no differences in spindle duration between the children with IH or subjective sleepiness and their controls.

**FIGURE 1:**
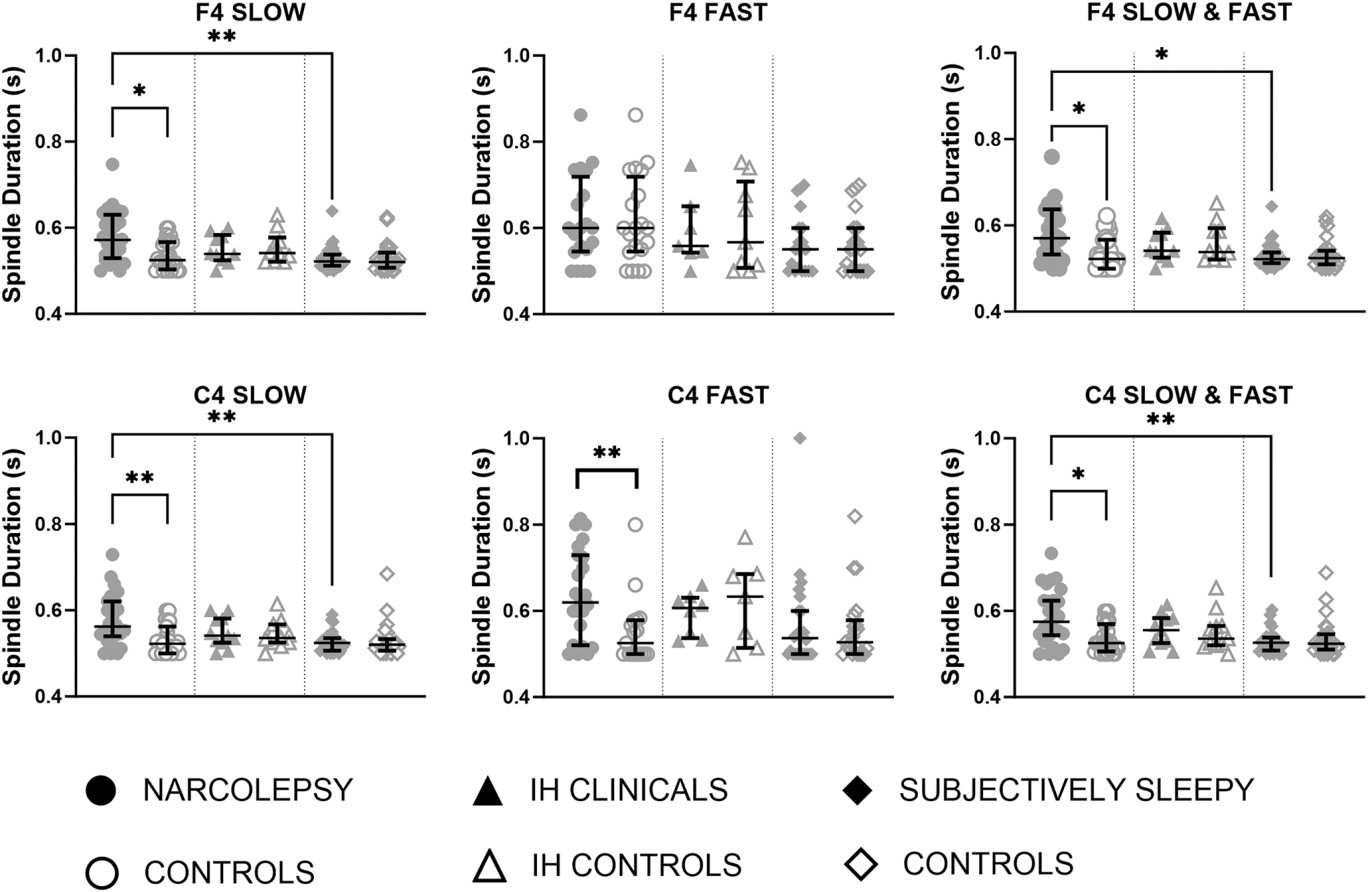
Comparison of sleep spindle duration in frontal (F) and centroparietal (C) regions for fast, slow and fast and slow spindles combined in children with narcolepsy, idiopathic hypersomnia and subjective sleepiness and their matched controls. Values are median and IQR. *p < 0.05; **p < 0.01.

Comparison of spindle density is presented in Figure 2. There were no differences in spindle density between the narcolepsy, IH or subjectively sleepy groups of children. In the children with narcolepsy C4 Slow spindle density was greater (median 0.127 vs. 0.038, *p* = 0.046) compared to controls. There were no differences in spindle density between the children with IH and their controls. In the children with subjective sleepiness C4 Slow (median 0.127 vs. 0.036, *p* = 0.030) and C4 Fast&Slow (median 0.142 vs. 0.048, *p* = 0.033) spindle density was greater compared to their controls.

**FIGURE 2:**
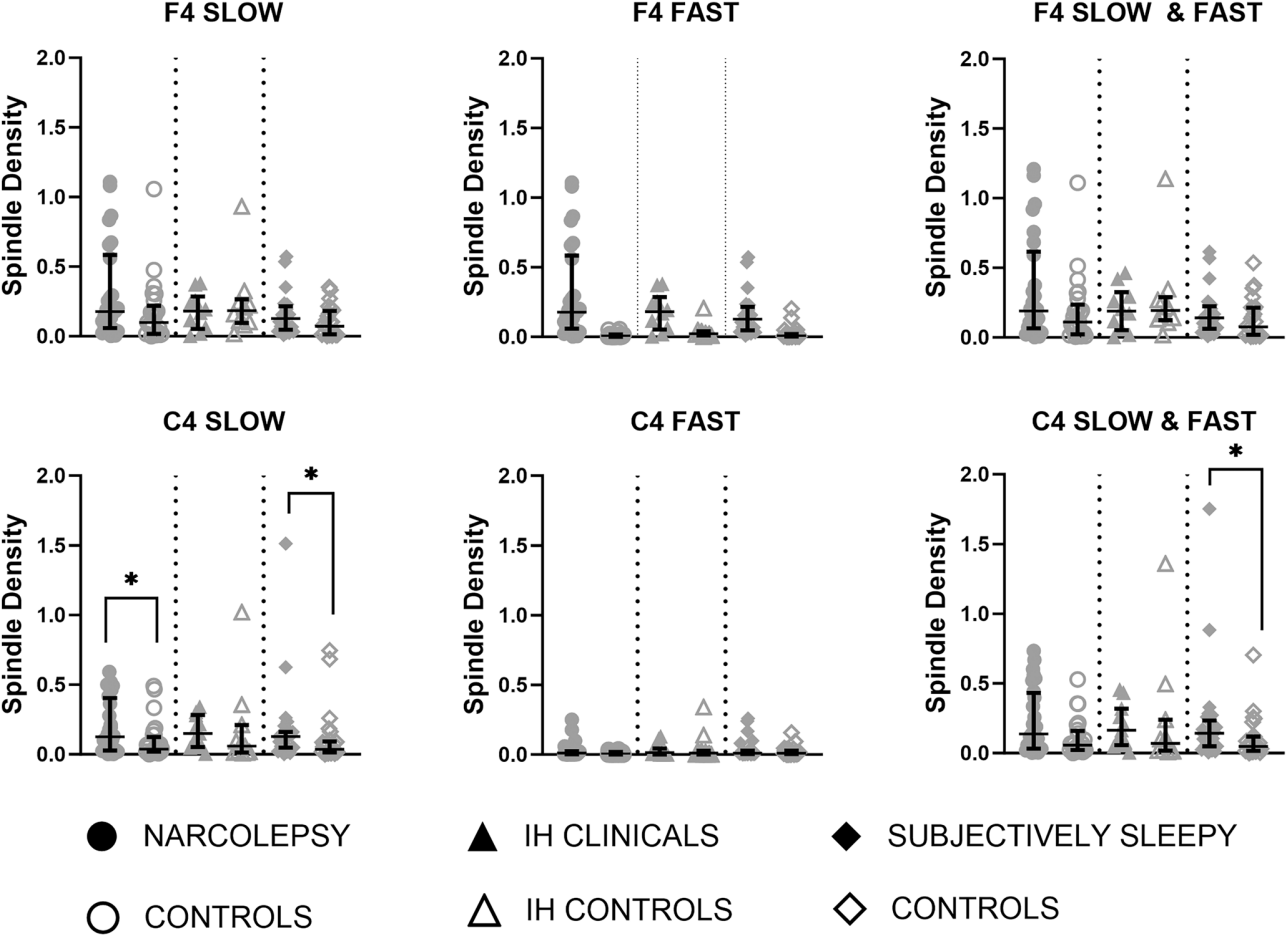
Comparison of sleep spindle density in frontal (F) and centroparietal (C) regions for fast, slow and fast and slow spindles combined in children with narcolepsy, idiopathic hypersomnia and subjective sleepiness and their matched controls. Values are median and IQR. *p < 0.05.

Comparison of spindle intensity is presented in Figure 3. There were no differences in spindle intensity between the narcolepsy, IH or subjectively sleepy groups of children. In the children with narcolepsy spindle intensity of C4 Slow spindles (median 0.070 vs. 0.012, *p* = 0.032) and C4 Fast&Slow (median 0.075 vs. 0.031, *p* = 0.047) spindles was greater compared to controls. There were no differences in spindle intensity between the children with IH and their controls. In the children with subjective sleepiness C4 Slow (median 0.066 vs. 0.019, *p* = 0.027) and C4 Fast&Slow (median 0.075 vs. 0.026, *p* = 0.024) was greater compared to their controls.

**FIGURE 3:**
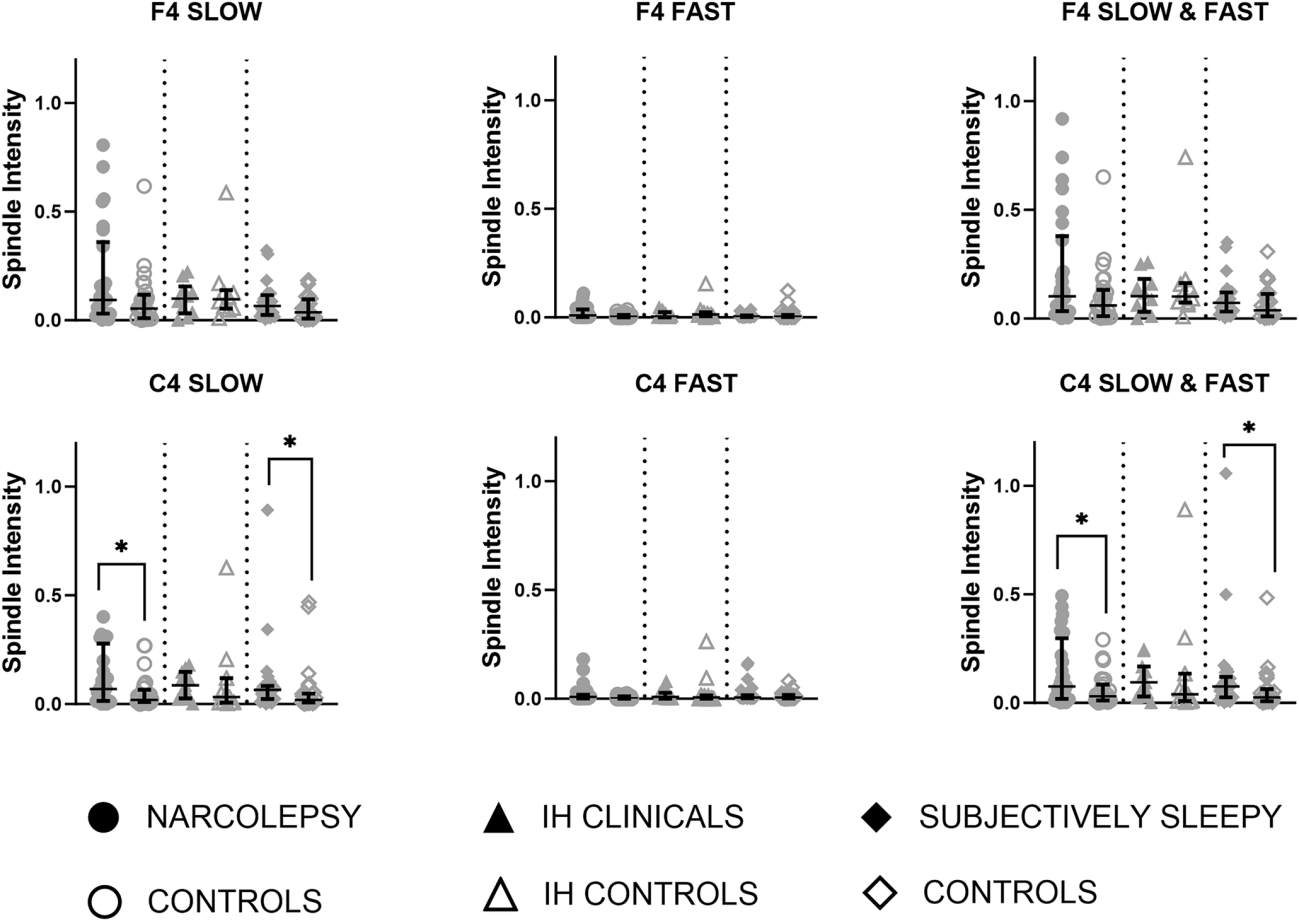
Comparison of sleep spindle intensity in frontal (F) and centroparietal (C) regions for fast, slow and fast and slow spindles combined in children with narcolepsy, idiopathic hypersomnia and subjective sleepiness and their matched controls. Values are median and IQR. *p < 0.05.

Slow spindle density was greater than Fast spindle density on both C4 and F4 electrodes in all groups (*p* < 0.001 for all). Slow spindle density was greater when recorded on frontal electrodes compared to central electrodes in the narcolepsy (median 0.178 vs. 0.127, *p* = 0.016) and narcolepsy control (median 0.099 vs. 0.038, *p* = 0.025) groups. In contrast, C4 spindle density was greater than F4 spindle density in the subjectively sleepy group (median 0.910 vs. 0.008, *p* = 0.004).

### 3.3 Differences in Spindle Indices Between Clinical and Control Groups in Each NREM Period Across the Night

Comparison of spindle duration between the clinical and control groups in each NREMP across the night is presented in Figure 4. Comparison of spindle density between the clinical and control groups in each NREMP across the night is presented in Figure 5. In the children with narcolepsy C4 Fast&Slow spindle density was lower in NREMP1 compared to NREMP4 (median 0.030 vs. 0.254, *p* = 0.027) and in the children with subjective sleepiness C4 Fast&Slow spindle density was lower in NREMP1 (compared to both NREMP3 [median 0.0364 vs. 0.135, *p* = 0.014] and NREMP4 [median 0.0364 vs. 0.114, *p* = 0.019]). Comparison of spindle intensity between the clinical and control groups in each NREMP across the night is presented in Figure 6. In the children with narcolepsy C4 Fast&Slow spindle intensity was lower in NREMP1 compared to NREMP4 (median 0.023 vs. 0.158, *p* = 0.025) and in the children with subjective sleepiness C4 Fast&Slow spindle intensity was lower in NREMP1 compared to both NREMP3 (median 0.018 vs. 0.072, *p* = 0.014) and NREMP4 (median 0.018 vs. 0.060, *p* = 0.022).

**FIGURE 4:**
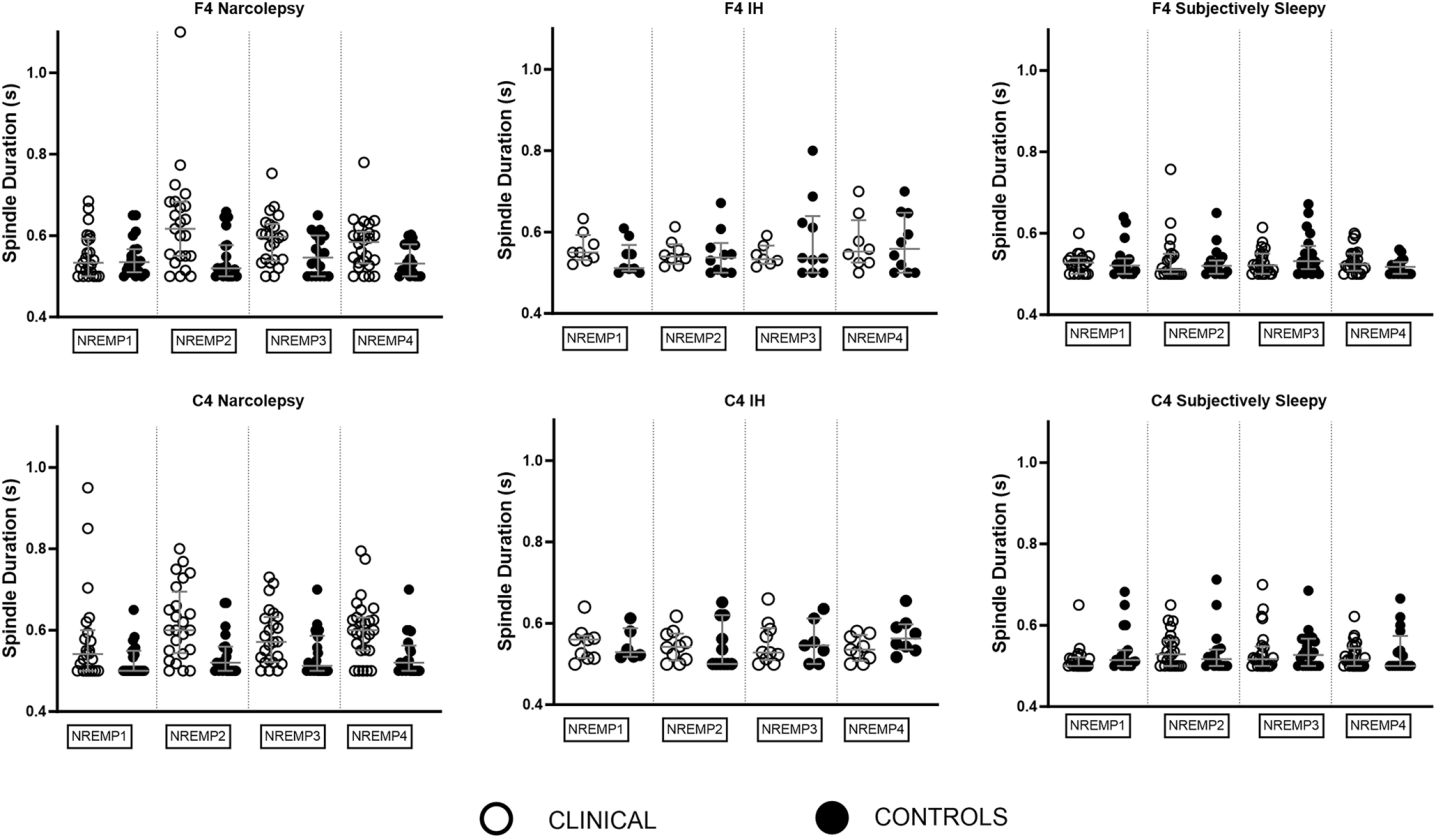
Comparison of spindle duration for slow and fast spindles combined between the clinical and control groups in each NREM period across the night. Values are median and IQR.

**FIGURE 5:**
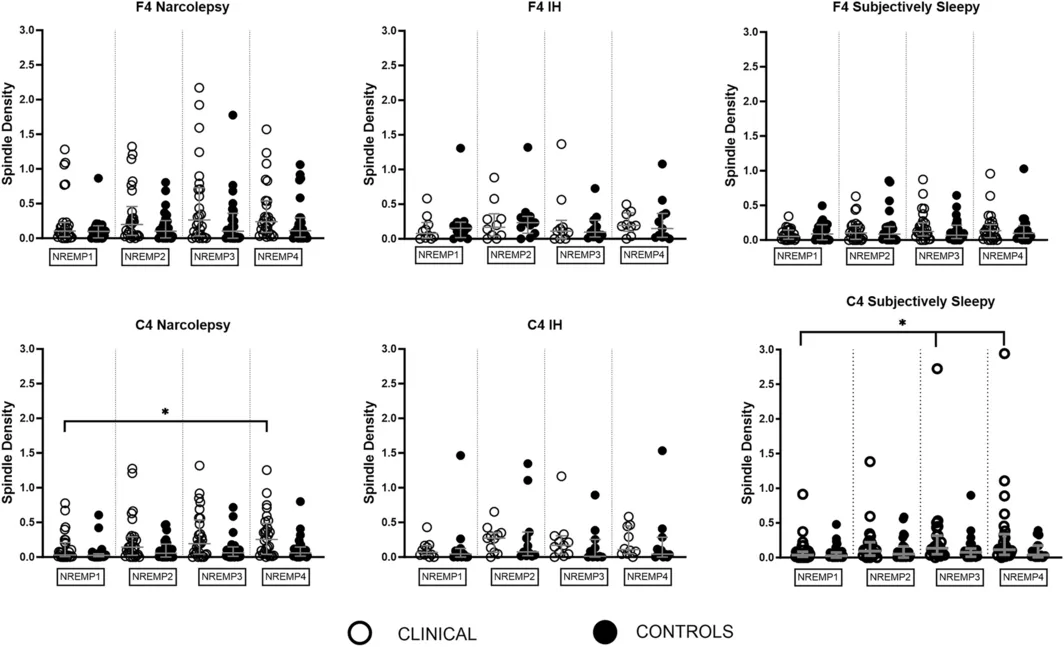
Comparison of spindle density for slow and fast spindles combined between the clinical and control groups in each NREM period across the night. Values are median and IQR. *p < 0.05.

**FIGURE 6:**
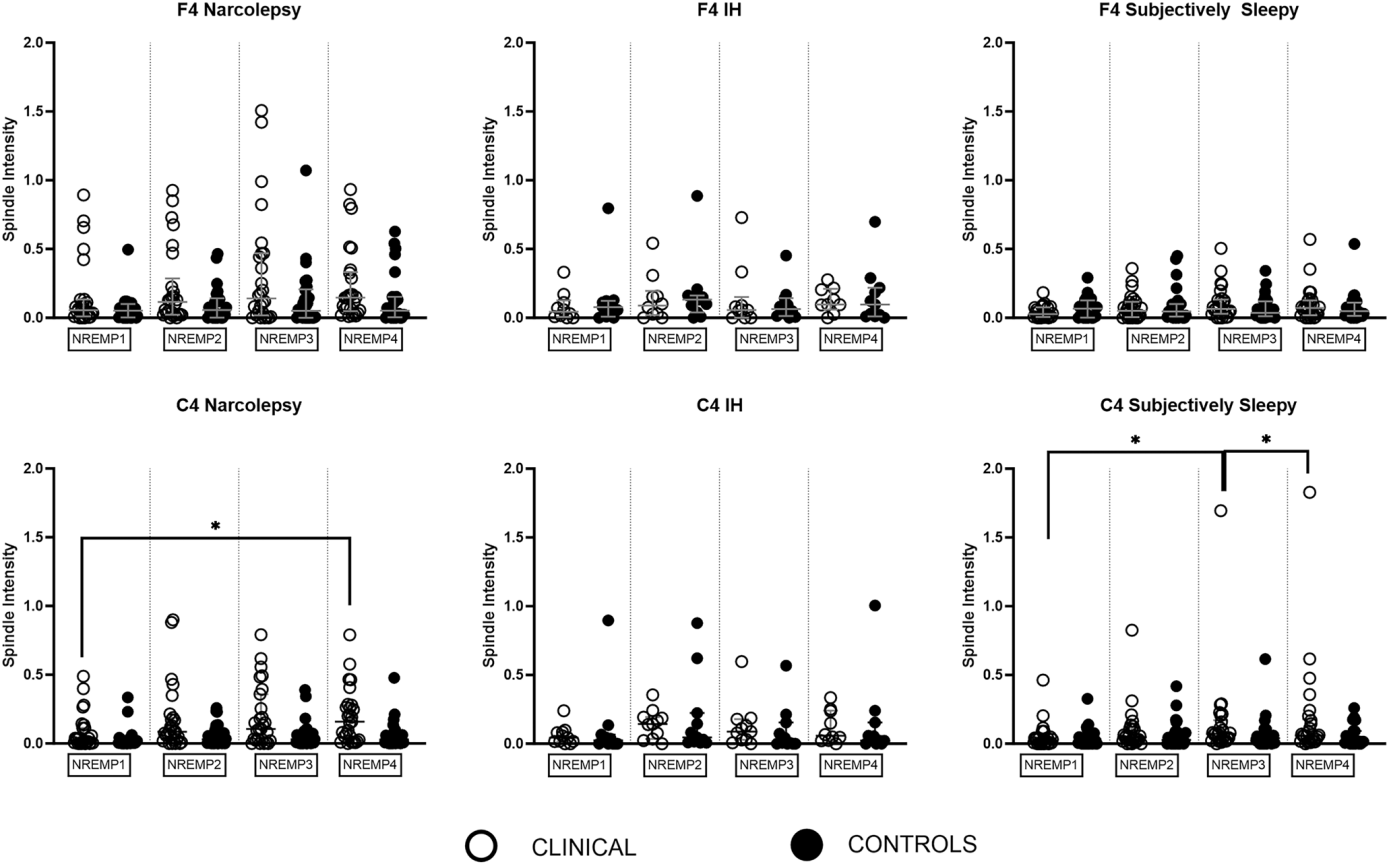
Comparison of spindle intensity for slow and fast spindles combined between the clinical and control groups in each NREM period across the night. Values are median and IQR. *p < 0.05.

### 3.4 Relationship of Spindle Activity to Severity of Subjective Sleepiness

The children with narcolepsy and IH had significantly higher scores for daytime sleepiness reported on the ESS‐CHAD compared to their matched controls as anticipated. There were no significant differences in ESS‐CHAD score between the three EDS groups or between the control groups. No significant correlations were seen between the ESS‐CHAD score and spindle number, density or intensity for either Fast or Slow spindles in either C4 or F4 in the clinically referred children (data not shown).

## 4 Discussion

Sleep spindle activity is a hallmark feature of healthy sleep and spindles are thought to raise the arousal threshold and prevent sleep disruption. Increased spindle density has been associated with narcolepsy and IH in some but not all studies in adults. This is the first study to compare spindle characteristics between children with narcolepsy, IH and subjective sleepiness and their matched controls. We found that spindle duration was longer in the children with narcolepsy compared to their matched controls and also compared to subjectively sleepy children who did not meet MSLT diagnostic criteria for a primary disorder of hypersomnolence. There were no differences in spindle duration between the children with IH or subjective sleepiness and their controls. In the children with narcolepsy and subjective sleepiness, spindle intensity of C4 Slow and C4 Fast&Slow spindles was greater compared to their matched controls. Spindle density and intensity of C4 Fast&Slow spindles were lower in the first NREMP compared to the fourth NREMP in the children with narcolepsy and lower in the first NREMP compared to both the third NREMP and fourth NREMP (*p* < 0.05 for both) in the children with subjective sleepiness. Our findings of differences in the subjectively sleepy group compared to controls, despite the fact that they did not meet the strict MSLT criteria, suggest that this group do differ in their sleep micro‐architecture. We have previously reported that many of these children develop narcolepsy later after a repeat sleep study (Nisbet et al. 2024). The absence of a relationship between the severity of subjective sleepiness (as measured by the ESS‐CHAD) and spindle activity supports the interpretation that the identified changes in spindle activity are related to the underlying diagnosis rather than the severity of sleepiness.

When we assessed spindles across the whole night we found that spindle duration, density and intensity were greater in the children with narcolepsy and those with subjective sleepiness compared to their matched controls with no differences identified between the children with IH and their controls. Only one previous study has reported spindle duration. In their study of patients with NT1 aged 8–21 years, Maski et al. also found that sleep spindles (12–15 Hz) detected automatically by a wavelet‐based algorithm averaged over the three central electrodes were longer compared to controls (Maski et al. 2025). However, they did not find any differences between groups for spindle density, amplitude, mean sigma power or mean frequency. The differences in findings between the studies could have been because our group of children with narcolepsy likely contained children with both NT1 and NT2 as cerebrospinal fluid measurements of hypocretin‐1 levels are not routinely performed in Australia and we could not definitively confirm the presence or absence of cataplexy for all children. The differences in methods of categorising spindles between the studies could also explain these differences. It is interesting to note that in a previous study in this same group of children examining EEG spectral power we did not identify any differences in sigma power, the frequency where spindles are located, between our groups (Plunkett et al. 2024), which may suggest that manual identification of sleep spindles is a more accurate, though time consuming, method of spindle detection.

We also identified that spindle duration was longer in the children with narcolepsy compared to those with subjective sleepiness. Comparison with previous studies of sleep spindles in patients with hypersomnolence is difficult as previous studies have used different clinical groupings. Bove et al. compared spindle density in adult patients with both narcolepsy and IH and found increased spindle density in the hypersomnia group compared to controls and when the hypersomnia group was divided into narcolepsy and IH, the IH group had the highest spindle density (Bove et al. 1994). The study by DelRosso et al. only compared patients with narcolepsy and IH, with no control group, and compared spindle number and spindle density during the first and last 50 epochs of N2 sleep and found higher spindle density in the patients with IH compared to the patients with narcolepsy in both the first and last 50 epochs of N2 sleep (DelRosso et al. 2014). The study by Christensen et al. compared spindle density between patients with NT1, NT2 and clinical controls who did not fulfil criteria for either NT1 or NT2 (similar to our IH group) and healthy controls and found no significant differences between groups (Christensen et al. 2017). In contrast to the studies by Bove et al. and DelRosso et al. we did not identify any differences in spindle indices between the narcolepsy and IH groups. This may have been due to the younger age of our subjects, as although both previous studies included a small number of children, the majority of the subjects were adults. Spindle density, particularly the percent of Fast spindles, increases with age through childhood with a peak at 12–14 years of age, before declining in late adolescence (Ricci et al. 2021). It may be that the reduced spindle density in the adults masked differences between the groups in the previous studies. It could also have been due to the smaller number of children in our IH group and the inter‐subject variability.

This was the first study to include a subjectively sleepy group, those who had a non‐diagnostic MSLT. Our finding that sleep spindles were longer in the children with narcolepsy compared with the subjectively sleepy group is interesting. It may be that the increased spindle duration in the children with narcolepsy reflects their increased sleep propensity, as sleep spindles play a primary role in protecting the sleeping brain from external sensory stimuli and have been used as markers of sleep integrity (Dang‐Vu et al. 2010; Dang‐Vu et al. 2015).

A unique feature of this study was that we divided spindles into Fast and Slow and also assessed spindles from both the centroparietal and frontal EEG leads, analyses which have not been reported before in studies of people with hypersomnolence. Slow spindles predominate in frontal cortical areas, originate in the medial frontal region, and are associated with increased activation of the superior frontal gyrus. Fast spindles are dominant over central and parietal areas, originate in the precuneus, and are associated with the activation of the hippocampus, medial frontal cortex and brain areas associated with sensory‐motor processing (Mölle et al. 2011; Schabus et al. 2007). In all groups of children, there were more Slow compared to Fast spindles recorded from both frontal and centroparietal regions. This finding suggests that the spindles type appears to be unaffected in children with hypersomnolence as this pattern has previously been reported by ourselves in a cohort of children with and without sleep disordered breathing (Shetty et al. 2023) and in studies in healthy non‐snoring children (Chatburn et al. 2013; Hoedlmoser et al. 2014).

In typically developing subjects, Slow and Fast sleep spindle density is highest at the beginning of the night and lowest at the end of the night (Alfonsi et al. 2019; Purcell et al. 2017). In contrast to these findings, when we examined total sleep spindle characteristics for Slow and Fast spindles combined across the night in successive NREM periods, we did not identify any differences in spindle number or duration; however, spindle density in C4 was lower in the first NREMP compared to the fourth NREMP, and spindle intensity was lower in the first NREMP compared to both the third and fourth NREMPs in the narcolepsy group, and both spindle density and intensity were lower in the first NREMP compared to both the third and fourth NREMP in the subjectively sleepy group. Consistent with these findings of the analysis of spindles across the whole night, we found that F4 spindle number was greater in the narcolepsy and subjectively sleepy groups in the third and fourth NREMPs compared to their matched controls, and that spindle duration was longer in the second and fourth NREMP in the narcolepsy group compared to controls. These findings are in contrast to previous studies which have identified that total spindle density decreased across the night in patients with narcolepsy (Bove et al. 1994; Christensen et al. 2017; DelRosso et al. 2014). These differences may have been due to the age of the populations studied, as spindle changes across the night are dependent on age, with younger individuals showing greater increases in Fast spindle activity and older individuals showing greater decreases in Slow spindle activity across the night (Purcell et al. 2017). It could also have been due to the different methods of measuring sleep spindles across the night. Bove et al. measured C4 spindle density in hourly periods across the night (Bove et al. 1994), while DelRosso et al. measured spindle density in the first and last 25 min (50 × 30s epochs) of the night, finding a greater density at the end of the night compared to the beginning in both narcolepsy and IH groups (DelRosso et al. 2014). These authors noted that the N2 epochs were often not continuous and were often interrupted by other sleep stages. Christensen et al. divided the night into sleep cycles defined as starting with a period of wake or NREM and ending with a period of REM or wake of at least 20 min duration, and found a trend for decreasing unspecified spindles (11–15 Hz), Fast and Slow sleep spindle density between the first and last sleep cycle in the healthy controls and NT1 patients (Christensen et al. 2017). It may also have been due to our combining Fast and Slow spindles for this analysis.

Our findings on sleep macro‐architecture were as would be expected, with children with narcolepsy having shorter sleep latency, REM latency and more time in REM sleep and less time in NREM sleep compared to their control group. These findings have been reported previously (Zhang et al. 2021). Our finding of increased TST in the children referred for assessment of EDS is likely due to the children being allowed to sleep longer (approximately an hour) in the morning to maximise sleep time before the MSLT compared to the controls who are discharged early in the morning.

The strengths of our study are that we individually matched each child referred for a MSLT with a control child without a history of EDS. We also included a group with undiagnosed EDS (negative MSLT) to investigate how they differed from those who had a diagnostic MSLT. In addition, all children, including controls, underwent overnight PSG so we were able to exclude children with obstructive sleep apnoea. Our spindle analysis was conducted by a single researcher (MS) in the interests of consistency in scoring of spindles. We must acknowledge the limitations of our study. We did not have cerebrospinal fluid measurements of hypocretin‐1 levels to distinguish between children with NT1 and NT2, and our narcolepsy group was likely a combination of the two, which may limit our ability to detect changes specific to NT1. As we are a tertiary referral centre for the state, not all children had clinical notes available at the time of testing to allow an accurate history of cataplexy to be determined. In addition, our sample size in the IH group was small.

In summary, our study identified that sleep spindles were longer in children with narcolepsy compared to children with subjective sleepiness and to matched controls. In addition, sleep spindles were more concentrated at the end of the night compared to the beginning of the night in both children with narcolepsy and those with undiagnosed subjective sleepiness compared to controls. Children with IH did not demonstrate these spindle findings, supporting a different disease entity. Our findings of differences in the subjectively sleepy group compared to controls despite the fact that they had a non‐diagnostic MSLT suggest that this group does have differences in their sleep micro‐architecture despite not meeting the strict MSLT criteria. This group likely includes young people who have evolving narcolepsy or IH but do not meet the current adult criteria for diagnosis but may be diagnosed after a repeat study (Nisbet et al. 2024). Understanding the microstructure of sleep in combination with clinical history may aid in distinguishing between groups of children referred for assessment of EDS, particularly those who do not meet strict diagnostic criteria for a primary disorder of hypersomnolence but did have abnormal MSLT results such as our subjectively sleepy group.

## Author Contributions

**Lisa M. Walter:** supervision, writing – review and editing. **Marisha Shetty:** investigation, formal analysis, writing – review and editing. **Nushrika Kadam Kishore:** investigation, formal analysis, writing – review and editing. **Lauren C. Nisbet:** writing – review and editing. **Margot J. Davey:** writing – review and editing. **Rosemary S. C. Horne:** conceptualization, funding acquisition, writing – original draft, writing – review and editing, supervision. **Georgina Plunkett:** formal analysis, writing – review and editing. **Gillian M. Nixon:** writing – review and editing.

## Funding

This work was supported by the National Health and Medical Research Council (1195453).

## Conflicts of Interest

The authors declare no conflicts of interest.

## Data Availability

The data that support the findings of this study are available on request from the corresponding author. The data are not publicly available due to privacy or ethical restrictions.
